# Using the Transient Response of WO_3_ Nanoneedles under Pulsed UV Light in the Detection of NH_3_ and NO_2_

**DOI:** 10.3390/s18051346

**Published:** 2018-04-26

**Authors:** Oriol Gonzalez, Tesfalem G. Welearegay, Xavier Vilanova, Eduard Llobet

**Affiliations:** MINOS-EMaS, Universitat Rovira i Virgili, Avda. Països Catalans, 26, 43007 Tarragona, Spain; oriol.gonzalez@urv.cat (O.G.); tesfalemgeremariam.welearegay@urv.cat (T.G.W.); eduard.llobet@urv.cat (E.L.)

**Keywords:** UV light, WO_3_ nanoneedles, NH_3_, NO_2_, gas sensor, dynamic operation

## Abstract

Here we report on the use of pulsed UV light for activating the gas sensing response of metal oxides. Under pulsed UV light, the resistance of metal oxides presents a ripple due to light-induced transient adsorption and desorption phenomena. This methodology has been applied to tungsten oxide nanoneedle gas sensors operated either at room temperature or under mild heating (50 °C or 100 °C). It has been found that by analyzing the rate of resistance change caused by pulsed UV light, a fast determination of gas concentration is achieved (ten-fold improvement in response time). The technique is useful for detecting both oxidizing (NO_2_) and reducing (NH_3_) gases, even in the presence of different levels of ambient humidity. Room temperature operated sensors under pulsed UV light show good response towards ammonia and nitrogen dioxide at low power consumption levels. Increasing their operating temperature to 50 °C or 100 °C has the effect of further increasing sensitivity.

## 1. Introduction

Metal oxide gas sensors have been attracting important research efforts because of their low cost and good sensitivity. Current research is addressing different drawbacks associated to metal oxides, such as the high temperature required to activate their response to gases, their lack of selectivity and, very particularly, their cross-sensitivity to humidity. Extracting information from the transient response of metal oxide gas sensors has achieved good results in the improvement of selectivity. Response transients can be obtained by producing step-changes in gas concentration [1,2,3,4,5,6], or by modulating/pulsing the sensor operating temperature [7,8,9,10,11,12]. Often, low-temperature operated metal oxide sensors suffer from slow response and recovery dynamics, which is not desirable in the continuous monitoring of ambient gases. Here, we explore a new way to create a transient in sensor response by pulsing a source of UV light to enable gas detection. In this case, the information extracted from the transient response is not used to improve selectivity, but to estimate gas concentration in a faster way, despite sensors being operated at low temperatures (compared to the standard operating temperatures of non-illuminated sensors) or even at room temperature. Moreover, the prospects of this procedure for reducing the influence of ambient moisture in sensor response are also investigated. Using UV light to activate the reactions taking place on the surface of metal oxide gas sensors has been considered by different research groups. For example, Sbeveglieri and co-workers explored, in the late 1990s, the UV activation of both SnO_2_ and In_2_O_3_ sensors operated at room temperature [13,14,15]. According to their research, excitation by means of UV light can affect, in different ways, charge carrier transport across the grain boundaries appearing in polycrystalline metal oxides. Namely, increasing the density of free carriers by photo generation, decreasing the intergrain barrier height, affecting the intergrain states charge and increasing the probability of tunneling through intergrain barriers, since the depletion layer width of adjacent grains is decreased. Moreover, light can change the occupancy of defects by both electrons and holes, thus affecting the absorption capacity of the metal oxide surface. On the other hand, illumination can also cause structural defects in the oxide lattice. In this case, these defects disappear when UV light is turned off, but with a long recovery period, that is usually temperature dependent. More recently, many other researchers have used UV light to activate gas detection at room temperature employing metal oxides such as TiO_2_ [16,17,18] or ZnO [19,20,21,22,23,24]. Trawka and co-workers [25] used a combination of heating and UV radiation to stimulate the interactions between WO_3_ and the surrounding gases, analyzing the effect of such interactions on the noise generated.

In other cases, UV light is used exclusively to clean the metal oxide surface, avoiding poisoning [26]. In fact, Trocino and co-workers [27] found that using UV light exclusively during the recovery period of room temperature operated In_2_O_3_ gas sensors, was more efficient than continuous UV illumination for measuring NO_2_. UV promotes desorption of NO_2_ and helps in regaining the sensor baseline. Recently, we introduced the use of pulsed UV light combined with thermal activation for measuring NO_2_ using In_2_O_3_ gas sensors [28,29]. Some preliminary results about the use of this technique in the measurement of NO_2_ employing WO_3_ sensors were presented in [30]. In this paper, we have explored further the approach of using pulsed UV to promote the response of WO_3_ nanoneedle gas sensors, either operated at room temperature or under mild heating (≤100 °C), in the presence of ammonia or nitrogen dioxide. The performance of this method in terms of response time, sensitivity, resilience to ambient moisture and power consumption is studied and compared against standard operation under constant heating.

## 2. Materials and Methods

The active layer of WO_3_ nano-needles was directly grown on ceramic alumina substrates using an aerosol assisted CVD. 50 mg of tungsten hexacarbonyl (W(CO)_6_) dissolved in a mixture of 15 mL of acetone and 5 mL of methanol were used as precursor. The substrates had printed Pt electrodes on one side and a Pt heater on the other side, allowing the sensor to be operated over room temperature. A piezoelectric ultrasonic atomizer generated an aerosol that was transported to the CVD reactor, where the substrates were located, by means of a flux of N_2_. The CVD reactor temperature was set to 500 °C. The growth process took about 15 min to complete. This resulted in the growth of randomly oriented, tungsten oxide nanoneedles of about 19 microns in length and 140 nm in diameter, fully coating the electrode area of the alumina substrate. The morphology and composition of the active nanomaterial was studied and confirmed by scanning electron microscopy (SEM) and energy-dispersive X-ray spectroscopy (EDX). These results are summarized in the Supplementary Materials. Then, a 2-h annealing at 600 °C in air was conducted to stabilize the gas sensitive films. Further details on the growth, morphology and composition of pure tungsten oxide nanoneedles can be found elsewhere [31].

Calibrated gas cylinders of nitrogen dioxide and ammonia balanced in synthetic dry air (1 ppm and 20 ppm respectively) were used in combination with a synthetic air gas cylinder to set the desired concentrations (dilutions) by means an arrangement of mass flow controllers. In these cases, the residual relative humidity in the measurement rig (at a room temperature of 25 °C) was found to be near 3%. In some cases, diluted samples were also humidified. For this purpose, once more using mass flow controllers, part of the dry air employed for sample dilution was passed through a bubbler to saturate it with humidity. This humidity-saturated flux was mixed with the sample cylinder, which enabled the desired concentrations of target gases under different humidity backgrounds to be obtained.

A Teflon chamber with an inner volume of 24 cm^3^ was used for characterizing the sensors. The chamber contains sockets allowing the connection of up to six sensors simultaneously. The chamber cover lid houses two UV LEDs, leaving a 12 mm spacing between the sensors and the LEDs. Since the radiation aperture of the LEDs is 120°, this distance assures a homogenous irradiation of the active layers. The LEDs employed were the model UVTOP320TO39FW from Sensor Electronic Technology Inc., Columbia, SC, USA (SETI) [32], with a maximum emitting optical power of 400 µW at 325 nm. In order to generate a transient in the sensor resistance when exposed, either to the gas to be measured or air, the UV diode was periodically switched ON and OFF, with a period set to 1 minute. This period was chosen in order to obtain a good signal to noise ratio, especially when the sensor resistance was high.

**Figure 1:**
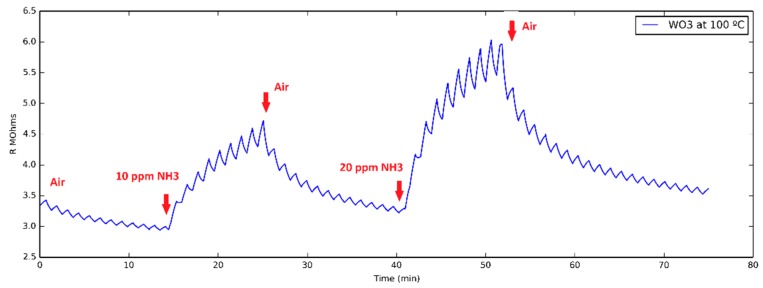
Typical evolution of the resistance of a WO_3_ nanoneedle sensor operated at 100 °C to two successive 10 and 20 ppm pulses of ammonia. The ripple in the resistance value is generated by a pulsed UV light (30 s ON and 30 s OFF) applied during the whole measurement process.

UV pulses result in the occurrence of a ripple in sensor resistance. This is reflected in Figure 1. This ripple is considered a transient signal in sensor response and the rate of resistance change both when the UV is ON or OFF have been found to give useful information for determining gas concentration [29]. Especially the fact that the rate of resistance changes when the light is OFF will be explored here, since it can be assumed to be closely dependent on the adsorption and reaction processes taking place on the gas-sensitive surface. Evaluating the rate in the period when the light is ON leads to similar information, but the signal is noisier.

This rate is computed as the local derivative of the resistance response curve, that is, the derivative when the UV diode is switched OFF is calculated according to the following expression:

(1) $$Rate\left(n\right)=\frac{R\left(n\right)-R\left(n-1\right)}{\Delta t}$$

where *R*(*n*) and *R*(*n* − 1) correspond to the final and initial value of sensor resistance for a period in which UV light remains switched off and Δ*t* is the duration of this period.

Although this methodology can be applied to sensors operated at room temperature, it will be applied as well to sensors under mild heating (≤100 °C). This is because metal oxides show, in general, higher response and faster response kinetics (e.g., higher resistance change rates) when heated than when operated at room temperature.

## 3. Results

### 3.1. Ammonia Detection

**Figure 2:**
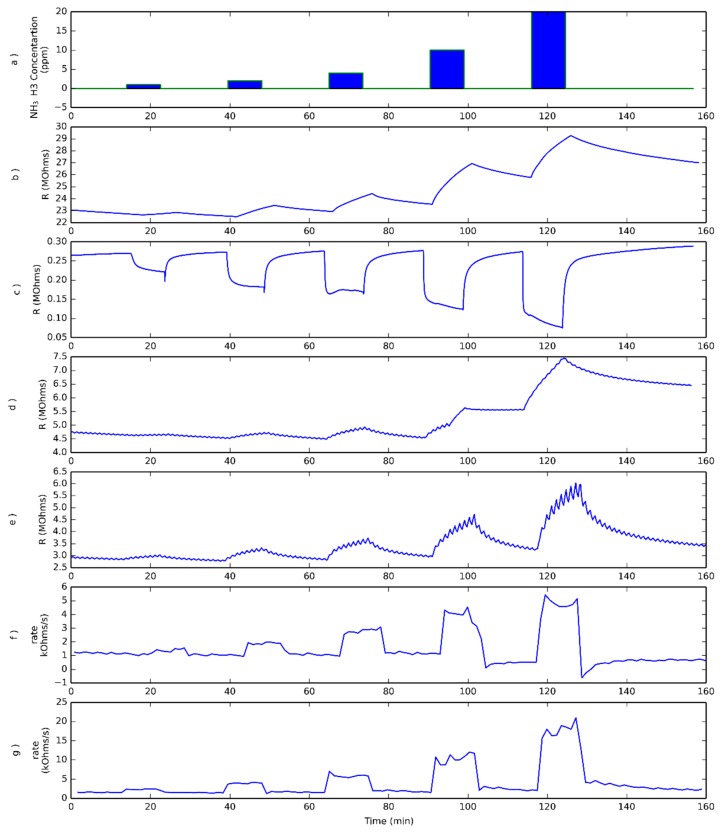
Increasing NH_3_ concentration pulses have been tested (**a**). Typical evolution of the resistance recorded in the dark for a sensor operated at room temperature exposed to ammonia pulses (**b**) equivalent to panel (**b**), but when the sensor is operated at 200 °C (**c**). Panels (**d**,**e**) show the responses to ammonia pulses described in the upper panel when the sensor is under pulsed UV light while being operated at room temperature or 100 °C, respectively. Lower panels (**f**,**g**) show the rates of resistance change when the pulsed UV light is OFF, as described in Equation (1), for a sensor operated at room temperature and at 100 °C, respectively.

In order to check the usefulness of this new methodology in the measurement of ammonia vapors, five different ammonia concentrations (i.e., 1, 2, 4 ppm, 10 and 20 ppm), were generated and delivered to the sensor test chamber (see Figure 2a). The response of the sensors operated under different conditions was recorded. As can be seen in Figure 2b, when a sensor was operated at room temperature without UV excitation, the changes in sensor resistance are rather small for the lower ammonia concentrations tested and slow, so baseline resistance is never regained. Moreover, as it is shown in Figure 2b, sensor resistance increases during ammonia exposure, instead of decreasing as one would expect for an n-type semiconductor sensor exposed to a reducing species. Figure 2c confirms that when the sensor is operated at 200 °C, its behavior corresponds to the expected one, i.e., sensor resistance decreases when exposed to ammonia. Additionally, when heated, sensor response is faster, allowing the sensor to reach a steady state when exposed to ammonia and to recover its baseline when exposed to air. Nevertheless, the power consumption to reach 200 °C is rather large: 1.2 W.

Subplots d and e in Figure 2 correspond to the response towards ammonia of a sensor operated under pulsed UV light at room temperature and heated to 100 °C, respectively. In those cases, power consumptions are 41 mW and 560 mW respectively. These figures, comprising the power to operate the LED and to heat the sensor, are significantly lower than the 1.2 W required in the previous case. In both cases, the response ripple caused by pulsed UV light is clearly superimposed to the resistance change caused by the exposure of the sensor to ammonia or dry air. From the comparison of subplots b and d in Figure 2, it is clear that the trends in resistance change due to ammonia exposure are quite similar in both cases and the sensor does not reach the steady state, nor recovers the baseline, being the main difference the ripple superimposed in plot d. Another aspect that can be observed is that the sensor electrical resistance under pulsed UV light is substantially lower than the one corresponding to the sensor just operated at RT. When the sensor operating temperature is raised to 100 °C, the resistance change due to gas exposure is faster than in the previous case, but not as fast as the response recorded when the sensor is operated at 200 °C, as one can expect, since increasing the operating temperature in MOX sensors results in a reduction in response time 

**Figure 3:**
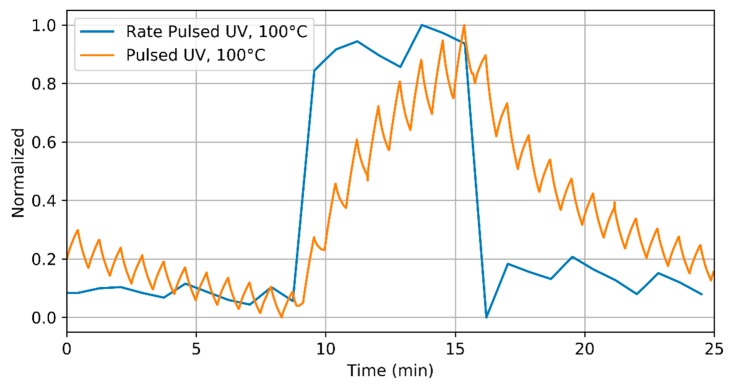
Detail of the sensor response to NH_3_, 5 ppm, when operated at 100 °C under pulsed UV light and the corresponding evaluated rate. Both appear normalized for clarity.

Finally, subplots f and g in Figure 2 show the rate of resistance change for sensors under pulsed UV light. The rates shown in subplots f and g were computed during the semi-periods in which the UV light was switched OFF and the sensor was operated either at room temperature or at 100 °C, respectively. It is clear from Figure 2 that the rate of resistance change shows a sudden increase when the sensor is exposed to ammonia, and that a quite stable plateau is attained, the value of which is related to ammonia concentration. When ammonia is removed from the ambient, this rate shows a sharp decrease as well. When the operating temperature is increased from RT to 100 °C, the value of the rate stabilizes at higher values, indicating that the adsorption-reaction process is faster. In order to compare the transient of the sensor resistance and the one corresponding to the evaluated rate, Figure 3 shows the resistance transient and the corresponding calculated rate both normalized. As shown in this figure, the rate shows a sharp change immediately after the sensor is exposed to the gas.

**Figure 4:**
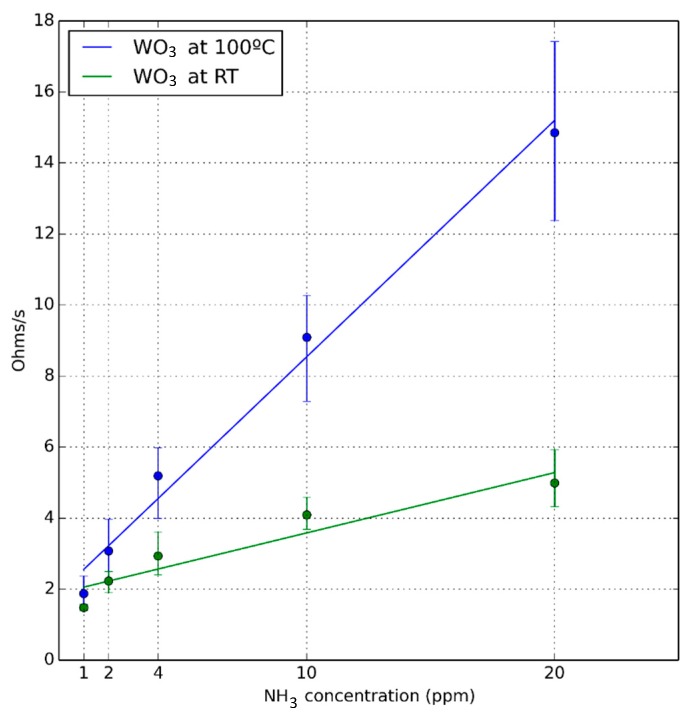
Calibration curves for the sensor operated at room temperature and at 100 °C. The response is the rate of resistance change as defined in Equation (1).

Figure 4 shows the calibration curves taking as response the rate of resistance change, evaluated when the sensor is operated under pulsed UV light at RT and at 100 °C. The error bars correspond to the maximum and minimum values obtained in the set of experiments. Even though the slope of the calibration curve (i.e., ammonia sensitivity) is higher when the sensor is operated at 100 °C, there is enough signal to reliably detect ammonia even when the sensor is operated at RT, with the consequent reduction in power consumption.

### 3.2. Nitrogen Dioxide Detection

**Figure 5:**
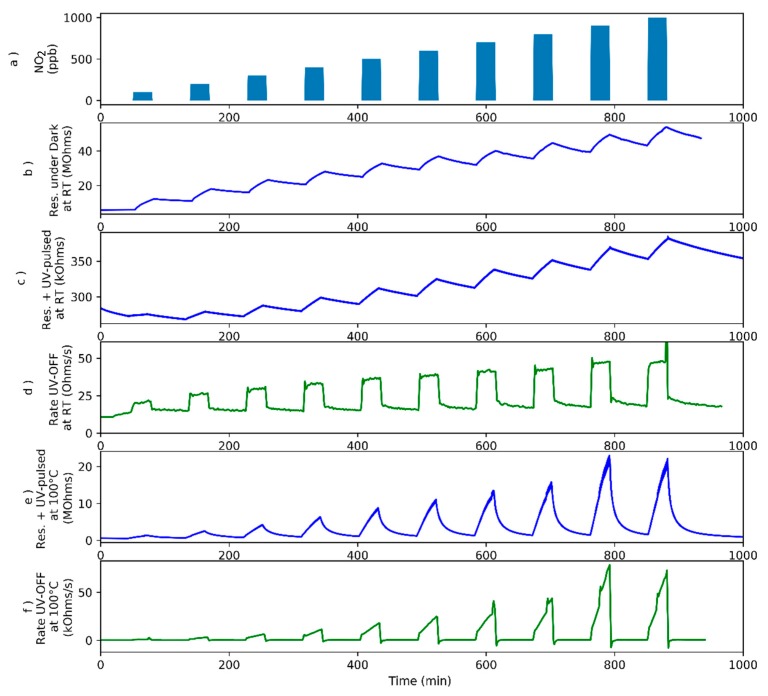
The sensor has been tested for several NO_2_ concentrations (**a**). Evolution of sensor resistance under dark conditions in response to successively increasing NO_2_ concentration pulses while the sensor is operated at room temperature (**b**). Evolution of sensor resistance under pulsed UV light in response to successively increasing NO_2_ concentration pulses while the sensor is operated at room temperature (**c**). Rate of resistance change evaluated when UV light is OFF as defined in Equation (1) while the sensor is operated at room temperature (**d**). Evolution of sensor resistance under pulsed UV light in response to successively increasing NO_2_ concentration pulses while the sensor is operated at 100 °C (**e**). Rate of resistance change evaluated when UV light is OFF as defined in Equation (1) while the sensor is operated at 100 °C (**f**).

In the case of nitrogen dioxide, 10 different concentrations were tested (100 to 1000 ppb measured at 100 ppb intervals), as can be seen in Figure 5a. In Figure 5b, the sensor working at room temperature without UV excitation shows a very slow response to nitrogen dioxide as can be also observed in the case of ammonia (Figure 2b). Figure 5c,d, show the changes in sensor resistance due to the combined effect of the exposure to nitrogen dioxide and pulsed UV light when the sensor was operated at RT and at 100 °C, respectively. Similar to what was observed for ammonia, sensor resistance does not reach steady state values during gas exposures nor is the baseline resistance regained during the cleaning phases. The resistance change rate, evaluated according to Equation (1), is depicted in Figure 5d,f, for a sensor operated at RT and 100 °C, respectively. When the sensor is operated at room temperature, once more, this response parameter shows a sharp increase upon exposure to nitrogen dioxide and reaches a plateau, which is clearly correlated to gas concentration. Furthermore, the rate returns to its baseline value during the cleaning phases. Nevertheless, when the sensor is operated at 100 °C the rate does not reach a completely stable value. It shows a sudden increase and then a drifting behavior with time.

**Figure 6:**
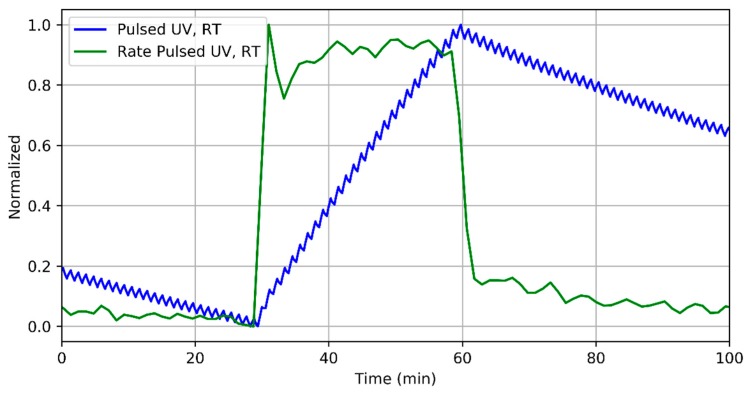
Detail of the sensor response to 400 ppb of NO_2_ when operated at room temperature under pulsed UV light and the corresponding evaluated rate. Both curves are normalized for clarity.

Figure 6 shows details of the normalized transient response (both electrical resistance and resistance change rate) to 400 ppb of NO_2_ when the sensor is operated at room temperature under pulsed UV light. As shown in this figure, the rate immediately reaches a stable value related to the gas concentration (this is similar to what was observed in Figure 3).

**Figure 7:**
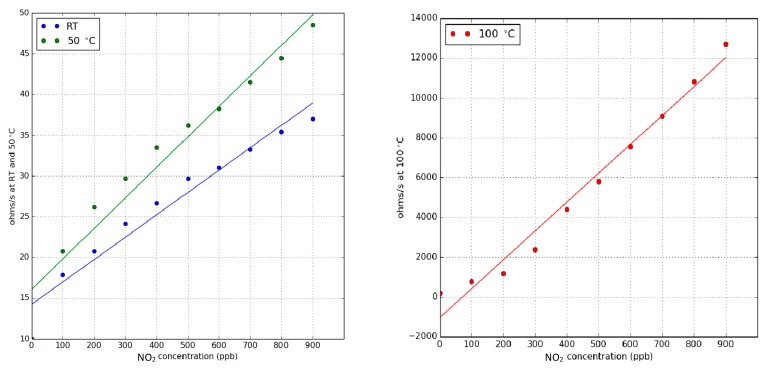
Calibration curves for nitrogen dioxide when the sensor is operated at room temperature, at 50 °C (**left panel**) and at 100 °C (**right panel**). The response is the rate of resistance change as defined in Equation (1). For any of the values reported in this figure, the uncertainty (variance associated to the diferent sensors tested and replicated measurements performed) remains below 15%. Reproduced from [30] © http://creativecommons.org/licenses/by-nc-nd/4.0/.

Figure 7 shows calibration curves for NO_2_ at different operating temperatures. Similar to the case of ammonia, heating the sensors has the effect of increasing the slope of their calibration curves (i.e., nitrogen dioxide sensitivity increases). However, the response and signal to noise ratio is good enough for reliably detecting nitrogen dioxide when sensors under pulsed UV light are operated at room temperature.

### 3.3. Humidity Effect

**Figure 8:**
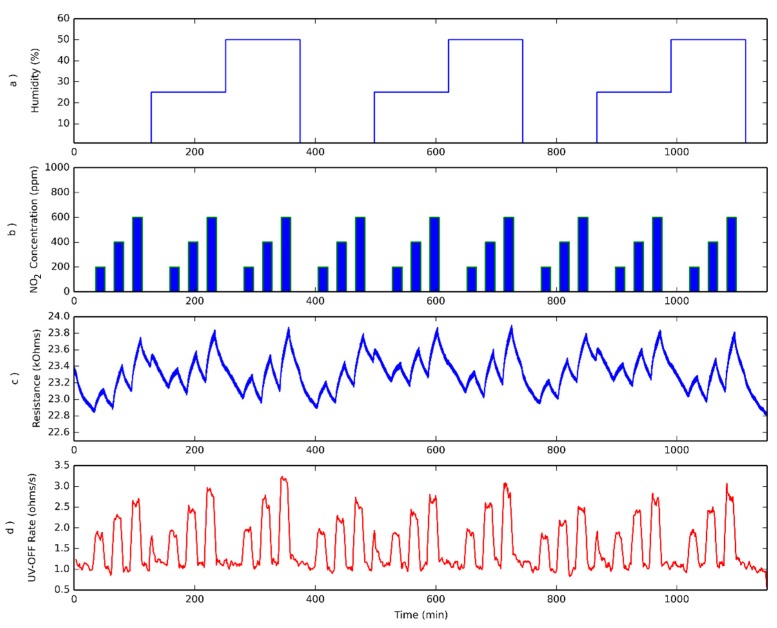
Effect of varying moisture levels on sensor response. The sensor has been characterized at three different humidity levels while operating at 50 °C (**a**), and at three different NO_2_ concentrations (**b**). Evolution of sensor resistance under pulsed UV light for the different humidity levels and NO_2_ concentrations shown in upper panels (**c**). Rate of resistance change evaluated when UV light is OFF, as defined in Equation (1), while the sensor is operated at 50 °C (**d**).

**Figure 9:**
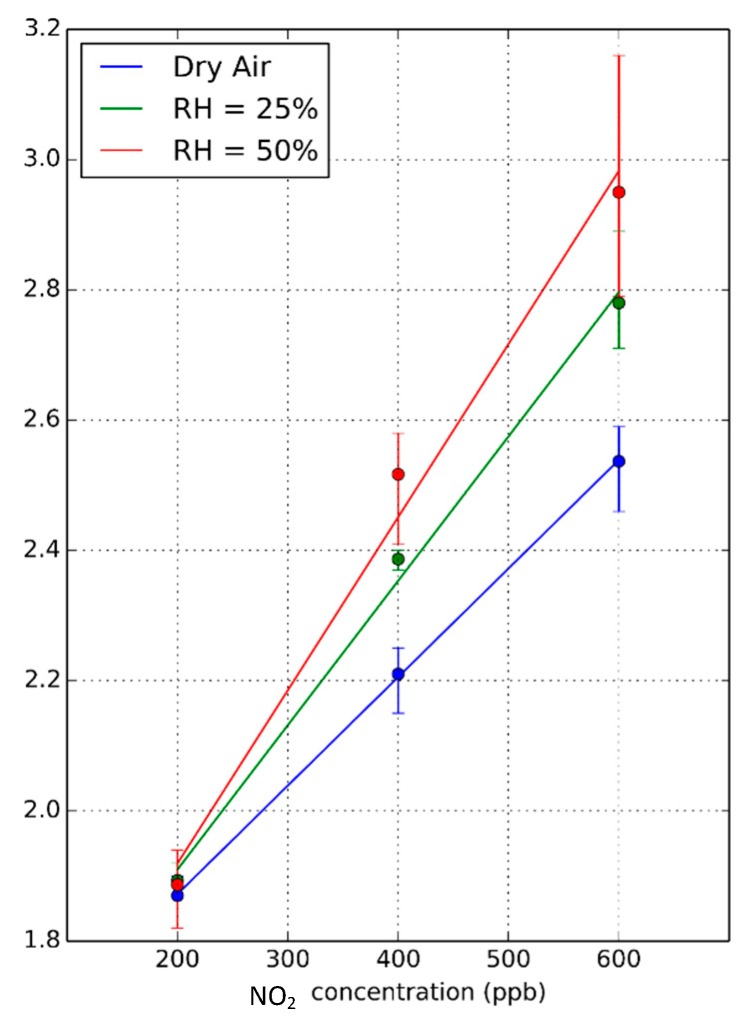
Calibration curves obtained from the rate of resistance change when pulsed UV light is OFF for three different humidity levels and for a sensor operated at 50 °C. Error bars correspond to the maximum and minimum values for the set of measurements gathered.

Additional measurements were performed at different humidity levels (dry air, 25% and 50% R.H.), considering three different NO_2_ concentrations (200, 400 and 600 ppb), as depicted in Figure 8a,b. In this case, an operating temperature of 50 °C was set, just to ensure that all measurements were performed at the same temperature and to avoid the occurrence of water condensation, since the water vapor generation system slightly increases the temperature of the gas flow input to the sensor chamber. As can be seen in Figure 8c, the sensor resistance reaches neither the steady state nor the baseline, as in the previous measurements in dry air. A small peak appears systematically when the system passes from dry air to 25% RH. This peak does not appear during the transition from 25% to 50% RH levels, which led us to associate this peak to a response when the sensor changes from dry to humid operation. Checking Figure 6d, where the resistance change rate is depicted, the three gas concentrations can be easily identified in spite of the different ambient humidity levels. An additional peak appears when passing from dry air to 25% RH, which is related to the previously described peak that appears in sensor resistance. Checking the calibration curves obtained for the three relative humidity levels (see Figure 9), it is possible to notice that, in fact, humidity has the effect of slightly increasing the sensitivity towards nitrogen dioxide. Therefore, the system is not immune to humidity, but the effect is quite low (sensitivity increases by about 10% when RH changes from 25% to 50%).

## 4. Discussion

According to the results reported by A. Giberti and co-workers [33], when WO_3_ is under UV light, the electrical resistance decreases due to a trapped surface charge lowering caused by two different phenomena. In the first case, when an electron-hole pair is generated near the surface, the electric field transfers the hole to the surface, recombining with an electron trapped there in O_2_^−^ form, causing as a result an O_2_ molecule to desorb. In the second case, the process consists of the photon being absorbed by an O_2_ molecule on the surface, allowing its desorption. Therefore, a new equilibrium between oxygen adsorption/desorption must be reached. In fact, photogenerated electrons can induce the adsorption of O_2_ molecules to form new O_2_^−^ ions, which are weakly bound to the WO_3_ [22]. On the other hand, when the light is switched off, the sensor surface recovers the initial equilibrium with the surrounding atmosphere, which is a very slow process.

We assume that while the UV diode is switched ON, UV light results in photo-generated charge carriers but also helps desorbing, at least partially, previously adsorbed species from the surface of the active layer. The combined effect of, at least, the two phenomena is probably the reason that causes the rate of electrical change in this period to be noisier. On the other hand, when the UV diode is switched OFF, the cleaned sensor surface may react with the species present in the surrounding atmosphere. During this period, the dynamics of this transient response are limited by the kinetics of adsorption, surface diffusion and chemical reaction, which are heavily affected by operating temperature [7].

Regarding the results obtained when the sensor is exposed to ammonia, it has been shown that when the sensor is operated below 200 °C, sensor resistance behaves in an opposite way to the one that would be expected for an n-type metal oxide in the presence of a reducing species. The electrical resistance of the sensor increases when exposed to ammonia rather than decreasing. This behavior can be observed whether the sensor is exposed to pulsed UV-light or not. This abnormal behavior for WO_3_ sensors when measuring ammonia at low operating temperatures (i.e., below 150 °C) has also been reported by other authors [34,35]. WO_3_ has also been reported to change from n-type to p-type behavior when operated at low temperatures and exposed to ethanol [36]. This change in behavior can be associated with the competition of two different mechanisms: the target gas can react both with the adsorbed oxygen ions and with the protons from adsorbed water molecules. The reaction with the adsorbed oxygen ions can modify the intrinsic conductivity of the metal oxide by changing the electron concentration, while the reaction with the water molecules adsorbed on the surface can modify the conduction of the surface water layer, which also contributes to the total conductivity. Nevertheless, this abnormal behavior is still the subject of debate and beyond the scope of this paper. In any case, according to the results exposed in the previous section, it is clear that, to be able to determine the gas concentration in a short period of time using the classical approach (i.e., based on the electrical resistance change), higher temperatures (and consequently higher power consumption) are required. Nevertheless, using the UV pulsed mode and evaluating the rate of change of the sensor resistance when the UV light is OFF, allows determining the gas concentration without reaching the steady state. It is important to point out that the time needed for the rate of resistance change to reach a plateau is very short. Since the value of this plateau can be evaluated well in advance, the sensor resistance reaches a steady state value; this enables for a fast determination of both ammonia and NO_2_ concentrations, even when the sensor is operated at low temperature, including RT. Therefore, this approach allows the determination of gas concentration for both chemical species in a shorter time, while keeping a reduced power consumption. Moreover, the effect of humidity in the measurements which is known to be of great importance for sensors based on pure WO_3_ [37,38], has a reduced impact when using pulsed UV light, probably due to the neutralization of hydroxyl groups by photogenerated holes while UV light is ON [39].

## 5. Conclusions

The electrical resistance transient of WO_3_ nanoneedle gas sensors under pulsed UV light has been analyzed. Pulsed UV light not only generates free charge carriers via valence to conduction band transitions, but also alters adsorption, desorption and reaction phenomena occurring at the gas-solid interface in metal oxide gas sensors. As a result, a modulation of the concentration of free charge carriers in the semiconductor is achieved. The rate of change in sensor resistance gives information about the concentration of the gas present in the environment. Using this approach, metal oxide nanoneedle sensors can be operated at significantly lower temperatures, even at room temperature, reducing the required power: For example, from 1.2 W in a sensor heated at 200 °C down to 41 mW in a room-temperature operated, pulsed UV light excited sensor. In addition, gas concentration can be estimated much faster than when the standard change in electrical resistance is used (i.e., when sensor is operated well above room temperature and, upon a change in gas concentration, it is necessary to wait until a static regime is achieved). Moreover, this approach has also been shown to be less affected by changes in ambient moisture. The methodology has been shown to be useful both for oxidizing (NO_2_) and reducing (NH_3_) gases.

## Supplementary Materials

The following are available online at http://www.mdpi.com/1424-8220/18/5/1346/s1. Figure S1: Sensor substrate used, Figure S2: SEM image of the WO_3_ nanoneedles conforming the active layer of the sensor, Figure S3: Typical EDX spectrum of a sensing layer, Figure S4: XRD spectrum for the WO_3_ nanoneedles, Figure S5: Raman spectrum for WO_3_ nanoneedles.
