# Supplementary material for: Using the Transient Response of WO3 Nanoneedles under Pulsed UV Light in the Detection of NH3 and NO2
Source: Sensors (Basel). 2018 Apr 26;18(5):1346. doi: 10.3390/s18051346 (PMC5982217; doi:10.3390/s18051346)
Supplement: Supplementary file 1 [file sensors-18-01346-s001.pdf]

SUPPLEMENTARY MATERIAL for

Using the transient response of WO3 nanoneedles under pulsed UV light in the detection of NH_3 and NO_2

Oriol Gonzalez, Tesfalem G. Welearegay, Xavier Vilanova* and Eduard Llobet

MINOS-EMaS, Universitat Rovira i Virgili, Avda. Països Catalans, 26, 43007, Tarragona, Spain.

[1] Affiliation 1; e-mail@e-mail.com
* Correspondence: xavier.vilanova@urv.cat; Tel.: +34 977 558 502

The active material has been deposited on the top of a pair of platinum interdigitated electrodes printed on a commercially available alumina substrate (Ceram Tech GmbH, Germany). This can be seen in Figure S1. The substrate includes a pair of Pt interdigitated electrodes (front side) and a Pt heating resistor (back side). Each electrode comprises 8 arms (300 µm in width) with an electrode gap of 300 µm. The electrodes cover an area of 2.5 × 2.5 mm^2. The heating element on the backside of the substrate had a resistance of 8 Ω at room temperature.

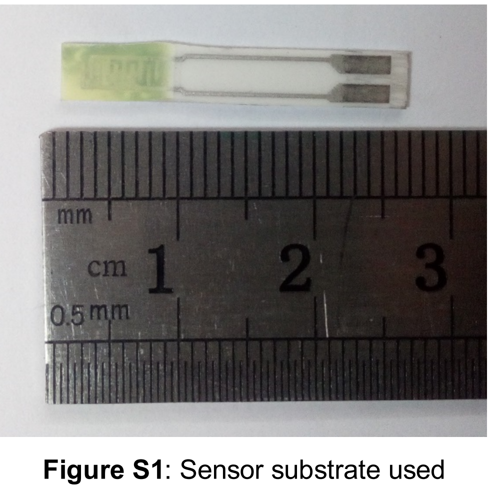

Figure S1: Sensor substrate used

The morphology and composition of the active nanomaterial was studied and confirmed by scanning electron microscopy (SEM), X-ray diffraction (XRD), Raman spectroscopy and energy-dispersive X-ray spectroscopy (EDX). Scanning electron microscopy images were acquired using a SU8020 Microscope from Hitachi and a JEOL 7600F field emission SEM. The image resolution was set to 512 × 384 pixels while the corresponding map resolution was set to 128 × 96 pixels, meaning a pixel

size of 0.01 µm in the image and of 0.05 µm in the map. EDX spectra were acquired using a microanalysis tool from Oxford Instruments. Raman spectrum was recorded using Raman FT-IR spectrometer from Renishaw equipped with a 514 nm laser. XRD was performed using a Bruker-AXS D8-Discover diffractometer with parallel incident beam and a 500 µm collimator in reflection mode.

Figure S2 shows typical SEM results for the nanomaterial grown on top of the electrode area of the alumina transducer. The film consists of a randomly oriented mesh of high aspect ratio nanoneedles (nearly 20 microns in length and 140 nm in diameter).

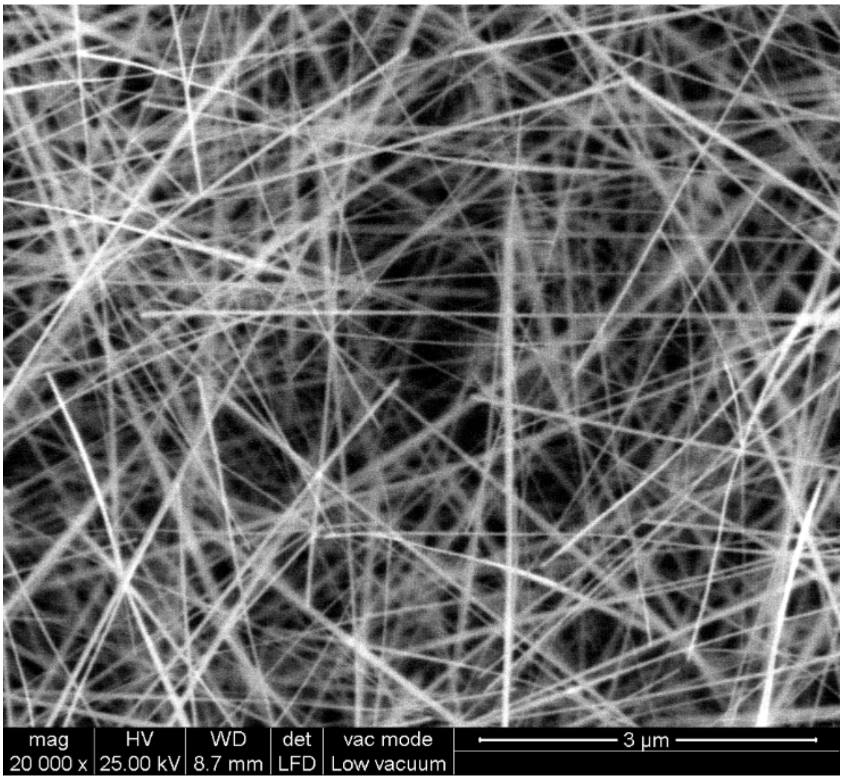

Figure S2. SEM image of the WO3 nanoneedles conforming the active layer of the sensor.

The results of the EDX analysis are summarized in Figure S3. These results confirm that nanoneedles consist of pure tungsten oxide and no other impurities are detected.

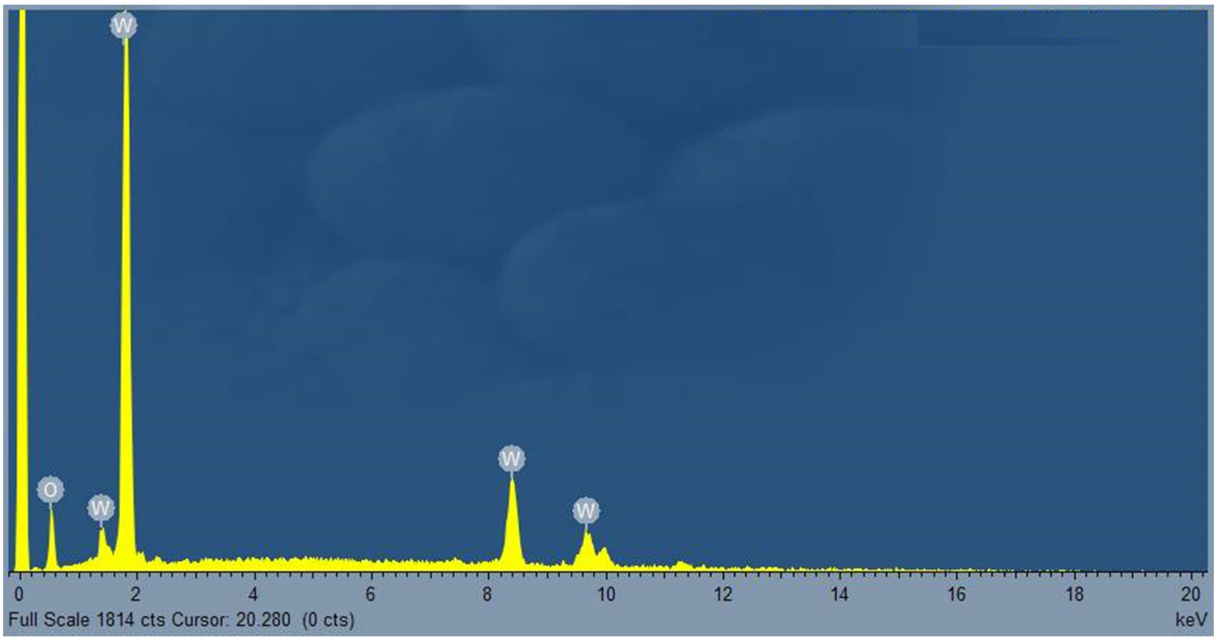

Figure S3. Typical EDX spectrum of a sensing layer.

The XRD Conventional 2theta diffractogram obtained for the AA-CVD deposited nanoneedles is shown in Figure S4. The spectra, according to JPDC 73-2177 card, corresponds to a WO3 P2/m, belonging to the monoclinic system, with a primitive cell and a space group 2/m with the cell parameters a= 18.32 Å, b=3.790 Å, c=14.04 Å with an angle β=115.20

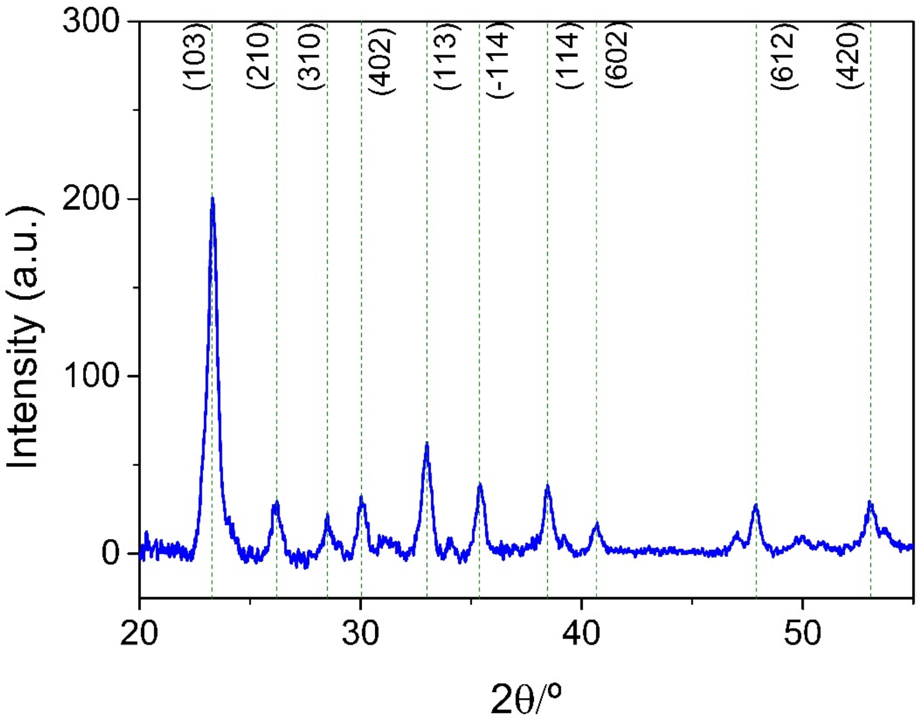

Figure S4: XRD spectrum for the WO3 nanoneedles

The Raman spectrum is presented in Figure S5, with a Band assignation according to Flores-Mena and co-workers [S1]

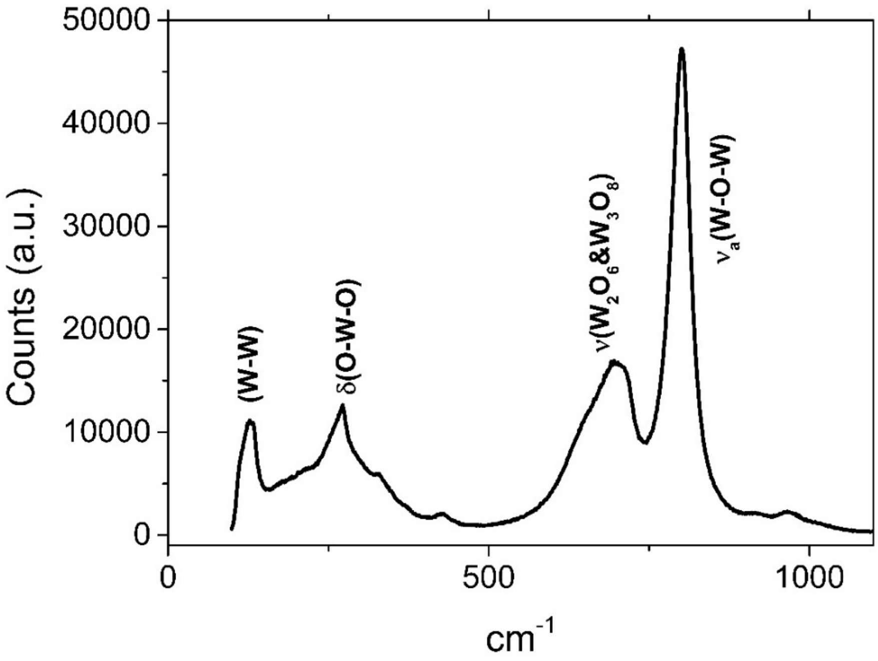

Figure S5: Raman spectrum for WO3 nanoneedles

[S1] E. Flores-Mena, J; Diaz-Reyes, J; Balderas-López, José, Structural properties of WO3 dependent of the annealing temperature deposited by hot-filament metal oxide deposition, Revista Mexicana de Fsica (2012) 58, 504-509.
